# Negative frequency dependent selection unites ecology and evolution

**DOI:** 10.1002/ece3.10327

**Published:** 2023-07-20

**Authors:** Mark R. Christie, Gordon G. McNickle

**Affiliations:** ^1^ Department of Biological Sciences Purdue University West Lafayette Indiana USA; ^2^ Department of Forestry and Natural Resources Purdue University West Lafayette Indiana USA

**Keywords:** balancing selection, biodiversity, coexistence, diversity, evolutionary ecology, genetic diversity

## Abstract

From genes to communities, understanding how diversity is maintained remains a fundamental question in biology. One challenging to identify, yet potentially ubiquitous, mechanism for the maintenance of diversity is negative frequency dependent selection (NFDS), which occurs when entities (e.g., genotypes, life history strategies, species) experience a per capita reduction in fitness with increases in relative abundance. Because NFDS allows rare entities to increase in frequency while preventing abundant entities from excluding others, we posit that negative frequency dependent selection plays a central role in the maintenance of diversity. In this review, we relate NFDS to coexistence, identify mechanisms of NFDS (e.g., mutualism, predation, parasitism), review strategies for identifying NFDS, and distinguish NFDS from other mechanisms of coexistence (e.g., storage effects, fluctuating selection). We also emphasize that NFDS is a key place where ecology and evolution intersect. Specifically, there are many examples of frequency dependent processes in ecology, but fewer cases that link this process to selection. Similarly, there are many examples of selection in evolution, but fewer cases that link changes in trait values to negative frequency dependence. Bridging these two well‐developed fields of ecology and evolution will allow for mechanistic insights into the maintenance of diversity at multiple levels.

## Graphical abstract

From genes to communities, understanding how diversity is maintained remains a fundamental question in biology. In this review, we relate negative frequency dependent selection (NFDS) to coexistence, identify mechanisms of NFDS (e.g., mutualism, predation, parasitism), review strategies for identifying NFDS, and distinguish NFDS from other mechanisms of coexistence (e.g., storage effects, fluctuating selection). We also emphasize that NFDS is a key place where ecology and evolution intersect.

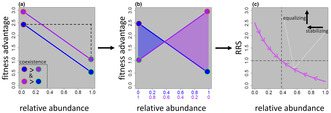

## 1 NEGATIVE FREQUENCY DEPENDENT SELECTION AND COEXISTENCE

Biological systems often contain a diversity of **entities** (see Glossary) that co‐occur in space and time. These diverse entities include genotypes, phenotypes, life‐history strategies, and species. But what maintains this diversity? Or perhaps more importantly: is this diversity actually maintained or are we simply witnessing some intermediate point in ultimately unstable dynamics? Even though **coexistence** is often considered as an ecological question at the community level, the maintenance of diversity at any level of biological organization can be conceptualized as an evolutionary question of coexistence. Here, we seek to review the importance of **negative frequency dependent selection (NFDS)** as the primary process that allows rare entities to increase in frequency and simultaneously prevents common entities from excluding rare ones—thus facilitating diversity and the coexistence of diverse entities (Brown, 2016; Grainger, Levine, & Gilbert, 2019; Vellend, 2016). Because coexistence is an equilibrium concept (i.e., a stable fixed point in a system of dynamical equations; May, 1973), and biological systems are rarely—if ever—at equilibrium, coexistence cannot be observed directly in a natural setting. Instead, only diversity and **co‐occurrence** can be observed, where co‐occurrence is a necessary, but not sufficient condition for coexistence (Blanchet et al., 2020). Thus, coexistence must be identified by combining observation, empiricism, and theory to infer dynamical trends in entity abundance over time. The study of NFDS provides one such avenue.

Many empirical examples of NFDS measure polymorphism within a single species (e.g., genotypes, phenotypes, or life‐history strategies). A classic example of negative frequency dependent selection occurs in nectarless (non‐reward giving) orchids, where individual plants produce either yellow or purple flowers. Experimental manipulations of the relative frequency of phenotypes revealed that the fitness of each color polymorphism declines with increased frequency due to pollinators associating flower color with a lack of reward (Gigord et al., 2001). Other examples include mating strategies within animals (Christie et al., 2018; Gross, 1991; Mokkonen et al., 2011; Sinervo & Calsbeek, 2006), color polymorphisms within damselflies (Takahashi et al., 2010), and defensive structures in plants (Goldberg et al., 2020). However, simply documenting NFDS is not sufficient for demonstrating coexistence, rather, empirical and observational data must be merged with theory to demonstrate coexistence over the long term (e.g., Christie et al., 2018; Takahashi et al., 2010).

**FIGURE 1:**
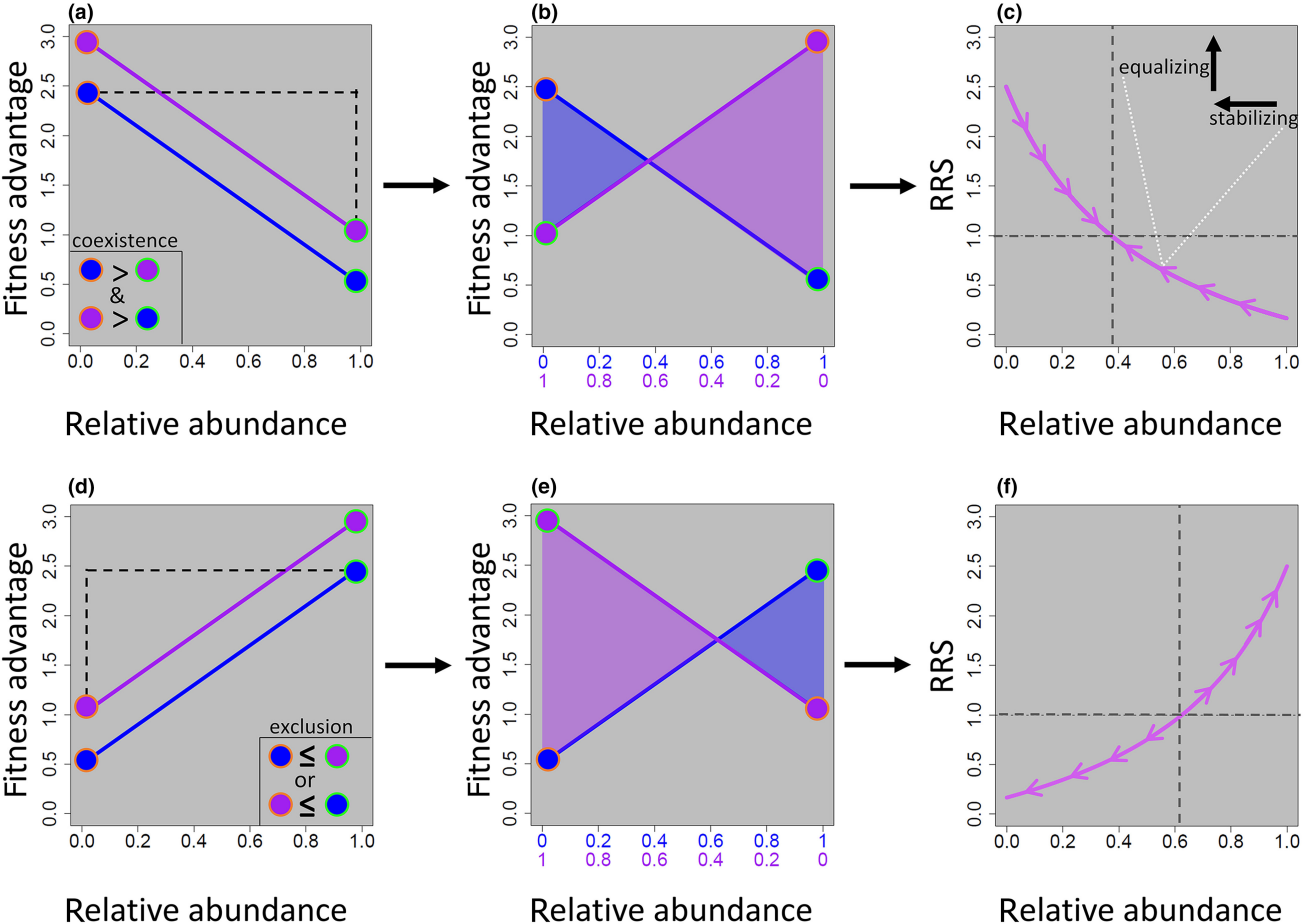
Negative frequency dependent selection occurs when an increase in relative abundance is accompanied by a decrease in absolute fitness (panel a). For two entities (e.g., genotypes) to coexist, the maximum fitness of the one entity must be greater than the minimum fitness of the other (dashed line). While panel (a) illustrates the NFDS relationships for two entities likely to coexist, notice that when one entity is at low abundance the other is at high abundance (relative abundances must sum to one) depicted with the dashed black line. It can be more intuitive to flip the abscissa for one species to visualize the dynamics of the population across various relative abundances (panel b). For coexistence to occur, both entities must have higher fitness at lower relative abundances (indicated with shading). Next, we can calculate the relative reproductive success (RRS) by dividing the absolute fitness of one entity by the absolute fitness of the other entity (panel c). Coexistence occurs when RRS decreases as relative abundance increases. Both stabilizing mechanisms and equalizing mechanisms draw entities towards the equilibrium, though entities may themselves rarely, if ever, be at equilibrium. By contrast, positive frequency dependent selection results in the increase of fitness with increasing abundance and movement away from the equilibrium at high or low relative abundance (panels d–f).

At a fundamental level, NFDS occurs when entities experience a per capita reduction in **fitness** with increases in **relative abundance** (Figure 1a,b). If the entities have small enough fitness differences, this leads to an evolutionary equilibrium that contains more than one entity (either within or among species), and an ecological equilibrium of non‐zero population sizes within each entity. Thus, as one entity becomes more abundant relative to other members, the accompanying reduction in fitness prevents exclusion, and as an entity becomes less abundant, the accompanying increase in fitness prevents extinction. While NFDS is a fundamental mechanism for the establishment and maintenance of coexistence, NFDS by itself is not always sufficient for continued coexistence as large fitness differences can prevent coexistence even in the presence of NFDS. For example, if the strength of NFDS is weak, and there is large environmental stochasticity, then a small increase in per capita fitness may not be sufficiently compensatory to prevent extinction at low abundance. Likewise, even if two entities exhibit strong NFDS, they cannot coexist unless the maximum fitness of entity 1 is greater than the minimum fitness of entity 2 and the maximum fitness of entity 2 is greater than the minimum fitness of entity 1 (Figure 1a). In other words, if one entity consistently has higher fitness than the other, it will always out compete the other entity, even in the presence of NFDS (i.e., the isoclines for fitness versus relative abundance must cross; Figure 1b). Consequently, the **relative reproductive success (RRS)** of both entities must decrease with increasing relative abundance and must cross the line y = 1 (Figure 1c). The steeper the slope of the RRS curve, the more likely for coexistence to occur. Thus, by studying abundance trajectories through time, or by directly manipulating relative abundance and measuring fitness outcomes, one can deduce the presence of NFDS and potentially entity coexistence.

## 2 MECHANISMS OF NFDS

At the most basic level, a mechanism that drives NFDS must decrease fitness (e.g., survivorship or viability, reproduction, or per capita rates of increase) as entities increase in relative abundance (Figure 1). Thus, many mechanisms of NFDS include biotic interactions.

Competition between two entities is perhaps the most straightforward mechanism of NFDS. When intra‐entity competition leads to stronger negative effects on population growth than inter‐entity competition, this process can drive the system to a stable equilibrium point of coexistence. For example, two different plant species with overlapping but different root morphologies may compete for limited nutrients (e.g., resources) found in the soil (Zepeda & Martorell, 2019). One species may better acquire nutrients at shallow soil depths, the other at deeper depths. Both species may initially do well at low densities. However, as the density of the shallow‐foraging species increases, additional individuals may have to grow longer roots to acquire nutrients over ecological time scales. This direct intraspecific competition decreases the per‐capita growth rate of the shallow‐foraging species, and the unequal interspecific competition prevents competitive exclusion and drives NFDS (Zepeda & Martorell, 2019). Nonlinear competition, where entities exhibit different, nonlinear responses to a shared resource, may also facilitate NFDS if as one entity increases in abundance, it simultaneously increases the competitive advantage of a different entity (Hartig et al., 2014).

Other interactions with a negative component (e.g., predation, parasitism) can also drive NFDS. For example, consider a predator–prey system where there is a mixture of bold and shy behavioral phenotypes among prey (Sih, Bell, & Johnson, 2004; Sih, Bell, Johnson, & Ziemba, 2004). Shy prey may have behaviors that initially allow them to avoid predation relative to bold prey. However, as the shy prey phenotype becomes more abundant, predators encounter shy prey phenotypes at a higher rate; increased encounter rates can result in predators capturing the more abundant prey at a higher relative rate. The rarity of bold phenotypes could then allow them to recover towards some stable coexistence between the two behavioral types. Prey switching, whereby predators actively choose more abundant prey (Kjellander & Nordstrom, 2003; Lack, 1954), greatly facilitates NFDS but is not a requirement for predation to maintain coexistence (Holt, 1984); small or subtle differences in predator preferences for prey can be sufficient to drive NFDS. Of course, predation may not always facilitate NFDS and a complete lack of predator preferences for prey or specialization on a single prey type could theoretically drive entities to local extinction; behavior, physiology, and evolutionary constraints can interact with abiotic factors (e.g., habitat structure) to determine whether predation imposes NFDS.

Perhaps the most ubiquitous manifestation of predator‐induced NFDS can be found in host‐pathogen systems. At a population level, pathogens may often evolve to exploit abundant genotypes; more abundant hosts translate to more abundant resources and higher fitness for the parasite. Consequently, abundant genotypes can eventually become burdened with a high infection load and allow less common genotypes to increase in frequency (Koskella & Lively, 2009). This pattern of NFDS can maintain host and pathogen genetic diversity through time. If hosts and pathogens (or predators and prey for that matter) are evolving in response to one another, NFDS can drive **red queen** dynamics (Van Valen, 1973, 1977). At a community level, pathogens may also evolve to exploit more abundant host species. More abundant hosts need not have more virulent pathogens to reduce abundance (though this is one possible mechanism) (Ewald, 1995), rather a higher cumulative parasite diversity and parasite load may interact to decrease per capita fitness. Conversely, when host species are at low abundance, single‐host pathogens may decrease in abundance due to limited density‐dependent transmission (Gao & Hethcote, 1992; Searle & Christie, 2021) and multi‐host pathogens may have higher fitness, similar to predators, by switching to more abundant hosts. The widespread prevalence of parasites and pathogens, coupled with their fast rates of evolution, suggest they may be among the most important drivers of NFDS. At a genetic level, NFDS can also be detected at immune‐related loci (e.g., major histocompatibility complex or MHC loci) (Bolnick & Stutz, 2017), which, while potentially challenging to distinguish from other forms of **balancing selection**, represents a possible avenue for future surveillance of NFDS (see section below).

Though less well documented, and less intuitive, positive interactions like cooperation (e.g., mutualism) can also lead to NFDS (Halloway et al., 2022). The logic is the same as competition, it requires each entity to have lower fitness due to intra‐entity interactions compared to inter‐entity interactions. Essentially any example of mutualism in nature can stand in here as long as there is a mixture of mutualistic and non‐mutualist types within a population (Carter et al., 2020; Markwei & LaRue, 1992; Yamamichi et al., 2020). The costs and benefits can be such that NFDS drives coexistence of cooperative and uncooperative entity types. For example, in a game theoretic model of plant‐microbe mutualism, it was shown that the number of competitors that defined the local neighborhood size could influence whether mutualism evolved, or whether mutualist and non‐mutualist genotypes actually coexisted via NFDS (Halloway et al., 2022). Unfortunately, the positive feedbacks in mutualism frequently lead to strange outcomes in many models (e.g., cheating evolves as the best strategy, or impossible infinite population sizes), and there is only a very narrow range of parameter space that creates NFDS in such models (Goh, 1979; McGill, 2005). Indeed, much of modern coexistence theory is undefined for mutualism (see Spaak & Laender, 2020). We flag the study of NFDS and mutualism as a key area that needs further research attention.

We have attempted to summarize the primary drivers of NFDS via the main modes of biological interaction, but reality can be more complex. Competition, parasitism, predation, and cooperation may all interact with varying degrees to drive NFDS. Additional mechanisms that facilitate NFDS may also exist. For example, sexual conflict can maintain color polymorphisms in damselflies (Takahashi et al., 2010), and this same mechanism may promote species coexistence. Additionally, negative frequency dependent sexual conflict can interact with differences in parasite tolerance to facilitate entity coexistence (Willink & Svensson, 2017). Competition, predation, and parasitism within and among genes, though rarely referred to in such terms, may also drive NFDS; gene duplication events, mobile elements, and co‐option may all serve to increase per capita fitness of rare genetic entities. Furthermore, NFDS need not operate continuously in space and time (Chesson & Warner, 1981) and may vary across environmental gradients (Grainger, Letten, et al., 2019). Populations, life‐history strategies, genes, genotypes, and species may drift or be pushed in and out of evolutionarily and ecologically neutral states. However, when entities are pushed to extreme relative abundances, NFDS is a key mechanism that can facilitate the coexistence of diverse entities.

## 3 IDENTIFYING NFDS

If coexistence is driven by NFDS, this phenomenon should be testable in that NFDS makes rare entities more abundant and common entities less abundant. Care must be taken when designing experiments that coexistence, not simply co‐occurrence, is detected and that treatments and controls meant to disentangle alternative mechanisms are carefully designed (Broekman et al., 2019; Grainger, Letten, et al., 2019; Siepielski & McPeek, 2010). One straightforward approach to identifying NFDS is with reciprocal invasibility experiments performed at multiple densities (Godwin et al., 2020; Hart et al., 2018). Such experiments should directly manipulate the relative abundances of two or more entities to examine frequency dependence. For example, entity 1 must be able to invade and persist when rare in a treatment where entity 2 is more abundant, and this should scale across densities. Conversely, entity 2 should be able to invade and persist when rare in a treatment where entity 1 is more abundant and this too should scale across densities (Kilgour et al., 2018). This reciprocal invasibility strongly implies the existence of an equilibrium coexistence point (e.g., Figure 1c). Paradoxically, the lack of reciprocal invasibility does not necessarily rule out NFDS as a mechanism of coexistence. For example, NFDS may operate under varied or longer time scales that preclude detection over the short duration of most experiments, experimental conditions may not match natural conditions required for NFDS (e.g., distribution and complexity of nutrients and symbioses that facilitate competition‐driven NFDS in the wild may be challenging to replicate experimentally), and insufficient statistical power and/or low effect sizes can all lead to failed detection of NFDS via experimental approaches.

Observational data may also be used to detect NFDS at multiple levels, though this may require fitting theoretical equations to infer population trends (Sinervo & Lively, 1996). On ecological time scales, differences in rates of relative lifetime reproductive success have been measured directly using intensive monitoring/tagging approaches (Goldberg et al., 2020), pedigree analyses (Christie et al., 2018; Fradgley et al., 2019), or indirectly with genetic methods such as calculating the effective number of breeders within each generation (Christie et al., 2012; Luikart et al., 2021). NFDS may also be detectable by identifying signatures of NFDS within and among genomes (Cavedon et al., 2019; Takeuchi et al., 2015). For example, Cavedon et al. (2019) found signatures of balancing selection related to different migratory behaviors among populations of caribou distributed along an environmental cline which, when coupled with strong signals of divergent selection between behavioral types, suggests a role for NFDS. Likewise, in spatially discrete host‐pathogen populations, immune‐related loci should be independently cycling for alleles that impart the highest per‐capita fitness (Agrawal & Lively, 2002). Thus, immune‐related alleles should be highly divergent among populations, yet show marked deviations from neutrality (e.g., high dN/dS ratio). Alternatively, populations with long, and highly overlapping generations (e.g., some species of trees), may show detectable signatures of NFDS. In these systems, the coupling of aging and genomic data could reveal generational shifts in allele frequencies correlated with effective population sizes. Cross referencing with highly‐conserved loci across taxa, these data could be employed within a theoretical framework to disentangle competing explanations for coexistence among entities.

Unlike manipulative experiments which can conclusively show that each entity can increase from rare to common and vice versa, observational approaches will be most effective when they combine data with theory (Christie et al., 2018; Godwin et al., 2020; Zepeda & Martorell, 2019). By combining theory with data, one can estimate parameters related to population interaction, and then project whether coexistence might be possible based on the observed demographic trends of the interacting entities within a population. Ideally, coexistence via NFDS that is identified from combining theory and observational data would be viewed as a testable hypothesis that is followed by a manipulative experiment, but such manipulations may not always be possible. We argue that in those cases, there are still important evolutionary insights that can be gained from fitting equations to observational data whereby different equations can provide insight into mechanisms that may drive NFDS. For example, in the case of competition among annual plants the Lotka–Voltera equations are probably the simplest approach and can be fit with as few as two parameters (Godwin et al., 2020; Spaak & Laender, 2020). However, there is a more mechanistic model of annual plant growth that includes fecundity, seed survival, germination rates, and competition among germinated growing plants (Godwin et al., 2020; Watkinson, 1980). The model has also been expanded to include the evolution of seed size as a trait under selection that influences seed bank dynamics (Rees & Westoby, 1997). Another approach would be consumer‐resource dynamics that can identify mechanisms directly related to resource consumption rates and exploitation competition (Chesson, 1990; Godwin et al., 2020; MacArthur, 1969). Thus, while the Lotka–Volterra approach is capable of phenomenologically identifying NFDS, the annual plant model would allow the separation of mechanisms related to seed bank dynamics versus competition among germinated growing plants, and the consumer‐resource model would allow the separation of mechanisms related to resource consumption. Though this is just one example, it will frequently be the case that some version of the Lotka–Volterra equations will be the simplest first order approximation of interactions among entities, and that higher order alternative models exist that may better serve to disentangle the many types of interaction.

## 4 ALTERNATIVES TO NFDS

Coexistence can occur via a variety of mechanisms that are not always mutually exclusive. Ecological mechanisms of coexistence often include various combinations of **stabilizing mechanisms**, which prevent exclusion and extinction, and **equalizing mechanisms**, which minimize fitness differences (Figure 1) (Chesson, 2000; Letten et al., 2017). An example of a stabilizing explanation for coexistence includes the storage effect (Facelli et al., 2005), where distinct life history stages, strategies, or behaviors can buffer individuals from present‐day hazards (e.g., competition, abiotic factors) (Abrams et al., 2013). For example, many plants produce seeds that can lie dormant for years where competition and mutualism are largely absent. The storage effect is not limited to plants and may be more ubiquitous than commonly appreciated. For example, sea lamprey (*Petromyzon marinus*) larvae can remain buried in sediment anywhere between 4 and 19 years before metamorphosing into parasitic adults (Renaud, 2011), the diapausing eggs of aquatic crustaceans can be revived after several hundred years (Hairston Jr et al., 1995), and salmon can return to spawn at various ages depending on ocean conditions (sometimes referred to as the “portfolio effect”) (Schindler et al., 2010). Storage effects can buffer against environmental and ecological variation and facilitate coexistence by preventing extinction and fostering continued co‐occurrence (Snyder & Adler, 2011). The key distinction between storage effects and NFDS is that storage effects, which can be fine‐tuned by natural selection and often result in a change in stage (e.g., seed to seedling, larva to juvenile) when conditions are improved, is that the emergence or transition of entities from “storage” need not result in per capita changes in fitness based on relative abundance (Ellner et al., 2016). For example, environmental conditions may drive large numbers of certain seed genotypes to germinate 1 year, but their higher relative abundance may not result in per capita reductions in fitness (Snyder & Adler, 2011). Nevertheless, the two concepts are clearly related, and one unanswered question is whether NFDS can drive the evolution of storage effects.

From an evolutionary perspective, coexistence is often explained via **fitness trade‐offs** where a fitness benefit associated with one function (e.g., size/age at reproduction) is correlated with a fitness cost of another function (e.g., survivorship declines with age) (Christie et al., 2018; Hereford, 2009; Stearns, 1989). However, we argue that fitness trade‐offs may not be a direct result of NFDS and need not directly facilitate coexistence. On the one hand, trade‐offs could simply be the result of stabilizing selection where there is an underlying unimodal relationship between trait values and fitness. The trade‐off in the unimodal event is that there is a benefit to increasing the trait value (e.g., size/age at reproduction), that peaks, but eventually there is a cost to increasing the trait value (e.g., survivorship) (Parker & Smith, 1990). In this case a trade‐off would not result in coexistence of multiple diverse traits, but rather it would result in selection for a single trait value. On the other hand, and for trade‐offs to appear to result in coexistence, there must be a multi‐modal relationship between fitness and trait values (Box 1). Such divergent selection might lead to speciation, or it might lead to the stable coexistence of diverse entities within a population. In this case, multiple entities could coexist due to NFDS and not due to the trade‐off, where common entities decline in fitness, and rare ones increase in fitness (e.g., Kilgour et al., 2018, Box 1).

BOX 1 **Ecology; putting evolution by natural selection back into Negative Frequency Dependence**

**FIGURE B1:**
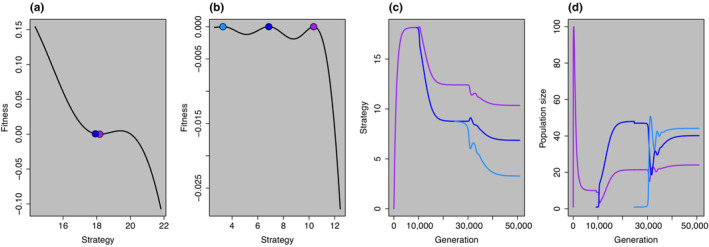
A simple example of NFDS, where we begin with one entity, and end in a world with three distinct entities which coexist through NFDS. The advantage here is that the model solution tells us how many entities (here modeled as life history strategies) coexist, rather than us telling the model a priori how many species we expect to coexist. To find the ESS of one life history strategy, or x life history strategies coexisting via negative frequency dependence, we developed a numerical simulation. The population began with two individuals with a strategy of $u_{i}=1$, and the ecological and evolutionary dynamics were allowed to unfold according to previously derived equations (Christie et al., 2018). When the strategy reached a minimum on the evolutionary landscape, which has been called an evolutionary branch point (Cressman et al., 2017), we allowed it to branch into two unique strategies, where the second strategy was phenotypically near to the original strategy. The rare new strategy could either go extinct or increase according to negative frequency dependent selection. This process continued until an ESS was found. The model is described in detail elsewhere (Carroll et al., 2009; Christie et al., 2018; MacArthur & Levins, 1967; Rees & Westoby, 1997).

Most ecologists will be quite comfortable with the concept of frequency dependence (Figure B1), particularly among species within a community. Indeed, from the Lotka–Volterra equations through to modern coexistence theory, there has been wide recognition that when diverse entities increase in frequency, coexistence requires self‐correcting mechanisms that prevent complete dominance and eventual competitive exclusion (Adler et al., 2007; Chesson, 2000; Germain et al., 2021; Hardin, 1960; Lotka, 1978; Volterra, 1926). Ecologists have shown that for a fixed number of entities, there is a limit to how similar entities can be before coexistence becomes impossible (Kraft et al., 2015; MacArthur & Levins, 1967). However, while less widely cited, almost as soon as ecologists identified limiting similarity, evolutionary biologists were quick to point out that the concept of limiting similarity skipped an important detail: evolutionary mechanisms create entity diversity, and ecologists often take that diversity for granted (Cressman et al., 2017; Rees & Westoby, 1997; Roughgarden, 1972). Why should there be more than one kind of entity in the first place? The question is an important one; if we take a fixed number of entities, with fixed similarities and differences, ecologists can routinely work out whether coexistence is possible (Hart et al., 2018; Kraft et al., 2015). However, the more vexing problem of predicting diversity from first principles has largely eluded ecologists. Moreover, the study of rapid evolution has shown that the assumption of fixed similarities or differences in ecological time is often invalid (Campbell‐Staton et al., 2017; Carroll et al., 2009; Hart et al., 2019). We argue that ecologists could benefit by more widely considering NFDS rather than simply NFD, and that such work could begin to work towards a more complete understanding of diversity, and mechanisms of coexistence. Such an approach could yield important mechanistic insights into trait and fitness variation at ecological timescales. Incorporating selection into ecological mechanisms has several distinct advantages: (1) the changes in populations through time have a heritable, genetic component (panel a), and (2) genetic changes in populations through time has implications for future responses to selection (panel b). All these changes are driven by the fitness landscape which is dynamic in time in models that marry ecological and evolutionary dynamics (panels C–D). The figure in this box shows results of a Lotka–Volterra competition model that includes trait evolution. Often competition parameters and carrying capacity are set as constant parameters, but here, in an eco‐evo model of NFDS, these parameters become functions of trait values. Carrying capacity is a function of the focal organism's trait values with the assumption that there are a smaller number of traits that are selected by the environment alone. This process generates stabilizing selection. The competition coefficients are a function of the focal species' trait values and their competitor's trait values with the assumption that like competes more intensely with like, a procedure that generates disruptive selection. Evolution proceeds along selection gradients, and speciation can occur in the case where evolution can go in more than one trait direction (panel a). The balance between stabilizing and disruptive selection ultimately determines the final diversity of the equilibrium community.

Evolutionary biologists also often invoke **fluctuating selection** as a driver of coexistence. However, when fluctuating selection is driven solely by environmental change it is not NFDS. The range of conditions required for environmentally‐mediated selection to continuously flip between alternative states in order to maintain coexistence over long‐term evolutionary time is improbable. When fluctuating selection is driven by entity abundance, it is, by definition, NFDS. Such fluctuating selection is just a special case of NFDS where high abundance can cause a sharp reduction in per capita fitness. When fluctuating selection is driven by stochasticity, multiple entities may co‐occur in time and space, but in the absence of some stabilizing mechanism, the fluctuations would ultimately lead to exclusion not coexistence over a long enough time horizon.

Evolutionary biology and ecology each have their own neutral theories (Kimura, 1983; Kimura & Crow, 1964; Rosindell et al., 2011). The neutral theory of molecular evolution focuses on molecular variation within and among species and posits that neutral processes such as mutation and drift, and their equilibria, are sufficient for explaining genetic variation within and among species. While numerous studies have argued in favor of neutral or selectionist viewpoints (Jensen et al., 2019; Kern & Hahn, 2018), we argue that both viewpoints can be correct. Indeed, ecologists have reconciled their neutral theory debate by recognizing that it is simply a special case of equal fitness and identical niches (Adler et al., 2007; Spaak & Laender, 2020). Coexistence requires NFDS, which in turn requires differential selection against common entities (Box 1). However, entities may be able to spend long periods of time in a state space governed by weak or even absent NFDS. Furthermore, NFDS is unlikely to select upon the entire genome and the targets of selection may vary over space and time. Thus, the observation that some portions of molecular variation may be principally governed by neutral processes, while others show evidence of various types of selection, does not directly conflict with NFDS, but neither do such theories provide a testable mechanism for explaining the coexistence of diverse entities.

Finally, if frequency dependent selection can be negative (NFDS), then it can also be positive (PFDS). **Positive frequency dependent selection** does not result in coexistence and often leads to exclusion. More specifically, PFDS can lead to priority effects, sometimes also called alternative stable states (Beisner et al., 2003; Gordon et al., 2015; Grainger, Letten, et al., 2019; Schreiber et al., 2019), where only one of two entities can exist within a population defined in both time and space. This occurs when neither entity can invade the other, thus creating a situation where whichever entity is found depends entirely on the evolutionary or ecological history (e.g., timeline of colonization or mutation events). It is important to recognize that while reciprocal invasion studies can be used to identify priority effects (e.g., Grainger, Letten, et al., 2019) and PFDS, observational data may simply appear as exclusion when PFDS operates.

## 5 NFD AND NFDS: UNITING ECOLOGY AND EVOLUTION

NFD and NFDS are a key area where ecology and evolution intersect. Particularly within the context of discrete species and coexistence, ecologists have long been thinking about negative frequency dependence. A better appreciation of NFDS (and not simply NFD) could greatly enhance understanding for the many cases where ecologists ordinate functional traits or behaviors along one or more axes. This includes bold‐shy continuum in behavioral syndromes (Sih, Bell, & Johnson, 2004; Sih, Bell, Johnson, & Ziemba, 2004), the slow‐fast continuum of life history traits (Bielby et al., 2007; Jones et al., 2008; or it's precursor r‐K selection; Pianka, 1970), the leaf economic spectrum (Wright et al., 2004), or the global spectrum of plant form and function (Díaz et al., 2022). When different species coexist due to different traits and trait values, this can manifest as NFD operating as limiting similarity (MacArthur & Levins, 1967). However, when multiple traits and trait values coexist within a species, this outcome can arise from NFDS. Take the bold‐shy continuum. One can always line individuals up along a single axis from high to low, large to small, bold to shy, and so on. Sometimes, this process will result in a unimodal distribution of trait values, where some intermediate value is likely under stabilizing selection, and the variation in bold and shy behaviors is just that, a standard deviation around a mean. However, if it can be shown that there is a multi‐modal distribution where more than one trait value is abundant, then NFDS is the more likely hypothesis. Testing for NFDS requires a reciprocal transplant study as described above. However, ecologists could make important theoretical gains by separating standard deviations around a single trait mean from true selection for diverse types within a population. An example of such a study was performed with fruit fly mutants that varied in aggression where, across multiple densities, non‐aggressive flies and aggressive flies survived better when rare (Kilgour et al., 2018).

Likewise, within the context of the maintenance of genetic diversity, evolutionary biologists have long been thinking about negative frequency dependent selection. But NFD and NFDS are fundamentally intertwined. It is impossible to have a per capita decrease in entity abundance (relative to other entities) that is not driven by selection (Box 1). Whether or not there is a response to that selection is a different matter, but that agent of selection is omnipresent in NFD and can change the fundamental ecological dynamics if rapid, heritable responses to that selection are possible. Similarly, many contemporary forms of selection may be couched within the broader framework of NFDS (Box 2). For example, positive selection that drives an allele from rare to common may on the surface appear as a response to selection that is occurring in a single direction that could ultimately decrease variation (both genetic and phenotypic). But over longer timescales, especially when accounting for metapopulation dynamics, or within the broader context of Red Queen dynamics, what appears as positive selection may also be NFDS and the corresponding changes in entity frequency may have longer‐term evolutionary consequences for coexistence. Thus, a substantial proportion of eco‐evo feedbacks may be driven by NFDS and we suggest that a broader appreciation of this intersection may further unite ecological and evolutionary theory.

BOX 2 **Evolution; putting Negative Frequency Dependence back into selection**

**FIGURE B2:**
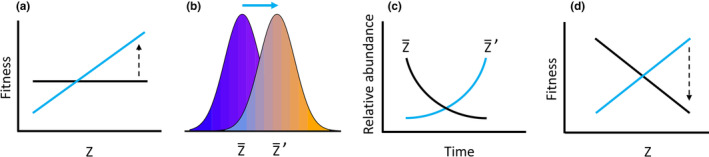
Fitness‐phenotype relationships are not static and environmental change may cause a shift in the fitness function (panel a) setting the stage for NFDS. This fitness differential can drive a shift in phenotype (panel b) that ultimately increases the relative abundance of the new phenotypes (panel c). Initially, the response to directional selection results in an increase in fitness that increases the relative frequency of new phenotypes (Z′). However, as the new phenotypes (and genotypes) become more abundant they can deplete the resources they have recently adapted to exploit and directly create a change to their fitness function (panel d) initiating NFDS.

Much work in evolutionary biology focuses on how new or rare entities increase in frequency (i.e., responses to positive or directional selection). By contrast, comparatively little empirical work has focused on negative frequency dependent selection. One reason for this discrepancy may lie in the fact that NFDS can be very challenging to identify. By contrast, directional selection results in a measurable shift in phenotype and the genetic underpinnings can be accurately identified with modern genetic tools (e.g., Barrett et al., 2019; Harder et al., 2020). But what is the expected outcome of NFDS? Within the framework of NFDS (e.g., Figure B2), one key metric is fitness. Fitness can be difficult to measure, much less define (Barker, 2009). Furthermore, fitness itself is the ultimate quantitative trait (Fisher, 1958; Rosenberg, 1978) and this reality makes it especially difficult to pinpoint relevant genes with measurable effects. Despite these challenges, we argue that there are several reasons for evolutionary biology to turn its focus towards NFDS. First, both positive and directional selection often appear to increase the frequency of new entities but may actually be encapsulated within the broader framework of NFDS. For example, consider a hypothetical example of NFDS—one driven by competition for a limited resource (e.g., food). Initially the fitness function may be flat. However, fitness‐phenotype relationships are dynamic in time and environmental change may increase the abundance of one food type causing a shift in the fitness function (panel a). This fitness differential drives a shift in phenotype (panel b) towards individuals that can exploit the more abundant food source and this response to selection ultimately increases the relative abundance of the new phenotypes (panel c). These shifts in phenotype occur with concomitant shifts in allele frequencies of loci governing the trait. Initially, the response to directional selection results in an increase in fitness that increases the relative frequency of new phenotypes (Z'). However, as the new phenotypes (and genotypes) become more abundant they deplete the abundant food resource and directly create a change to their fitness function to favor the original food resource (panel d). Over the short term, this process would (correctly) be identified as directional selection and has even been suggested as nascent speciation. Over a longer term, however, this NFDS (driven by competition) maintains phenotypic and genetic diversity within a population. More importantly, this example illustrates that scale and bias can interact to obscure the detection of NFDS and that NFDS may be more ubiquitous than is presently appreciated.

## 6 CONCLUDING REMARKS

Understanding the mechanisms responsible for the maintenance of coexistence within and among entities is fundamental to diverse ecological and evolutionary disciplines such as invasion biology, disease ecology, population genetics, and conservation biology. In this brief essay we suggest that NFDS is not only a primary mechanism for ecological coexistence, but also necessary for the long‐term coexistence of diverse entities such as genotypes, life history strategies, and species through evolutionary time. Nevertheless, there are still many unanswered questions regarding the ubiquity, testability, and specific mechanisms by which NFDS operates. For example, (1) How do anthropogenic disturbances and declines in biodiversity affect NFDS? (2) How does NFDS operating at one level (e.g., maintenance of genetic diversity) interact with NFDS operating at a different level (e.g., species coexistence)? (3) What are the genomic signatures of NFDS at multiple levels? Answers to this question could pave the way for practical approaches for detecting NFDS within and across taxa. (4) How does NFDS scale to N interacting entities? Most theoretical and empirical studies have focused on two to three interacting entities, but if NFDS maintains coexistence within entire ecological communities, general theories will be needed. Answers to these questions will go a long way to solidifying the foundational framework that our current understanding of NFDS is built upon and may shed light on how diverse entities coexist through space and time.

## Glossary

**Balancing Selection**: Various forms of selection that maintain entities (often used in the context of alleles) at higher frequencies than expected by genetic drift alone. Two common mechanisms include heterozygote advantage and NFDS.

**Coexistence**: when selection operates simultaneously on several levels of organization at the same time, often favoring cooperation at the group level and exploitation or cheating at the individual level.

**Co‐occurrence**: Two or more genotypes, phenotypes, or species observed to occupy overlapping boundaries in space and time. Co‐occurrence is necessary, but not sufficient, for coexistence.

**Entities**: Any diverse forms of genes, genotypes, phenotypes, life‐history strategies, behavioral types, or species.

**Equalizing mechanisms**: Any mechanism that serves to equalize fitness among entities.

**Fitness**: The average life‐time reproductive success of an individual; often considered as the product of viability (survival) and fecundity (reproduction). In many models of coexistence this parameter is captured as the per‐capita population growth rate at the genotype level.

**Fitness trade‐offs**: Occurs when a fitness benefit associated with one function (e.g., size at reproduction) is correlated with a fitness cost of another function (e.g., survivorship).

**Fluctuating selection**: Selection that changes direction, with respect to the sign of the change in mean trait value, through space or time. The change in direction can be mediated by either biotic or abiotic conditions (i.e., it can be stochastic).

**Negative frequency dependent selection (NFDS)**: The per capita reduction in fitness associated with increasing abundance relative to other entities.

**Positive frequency dependent selection (PFDS)**: The per capita increase in fitness associated with increasing abundance relative to other entities.

**Red Queen**: An evolutionary hypothesis which posits that, to survive, entities must evolve in response to constantly evolving, but opposing entities.

**Relative abundance**: The abundance of one entity relative to at least one other entity. For mathematical simplicity, theoretical examinations of coexistence often focus on two entities.

**Relative Reproductive Success (RRS)**: The lifetime reproductive success of one entity relative to another. The entity with higher reproductive success is typically used as the denominator and an RRS equal to 1 means that both entities have equal fitness.

**Stabilizing mechanisms**: Any mechanism that serves to stabilize the relative abundances of entities.

## AUTHOR CONTRIBUTIONS

**Mark R. Christie:** Conceptualization (equal); project administration (lead); writing – original draft (equal); writing – review and editing (equal). **Gordon G. McNickle:** Conceptualization (equal); writing – original draft (equal); writing – review and editing (equal).

## Data Availability

No new data were generated in the writing of this manuscript.
